# Quality Assessment of Fresh Meat from Several Species Based on Free Amino Acid and Biogenic Amine Contents during Chilled Storage

**DOI:** 10.3390/foods7090132

**Published:** 2018-08-25

**Authors:** Mehdi Triki, Ana M. Herrero, Francisco Jiménez-Colmenero, Claudia Ruiz-Capillas

**Affiliations:** 1 Ministry of Public Health, P.O. Box 42, Doha, Qatar; 2 Department of Products, Institute of Food Science, Technology and Nutrition, ICTAN-CSIC, Ciudad Universitaria, 28040 Madrid, Spain; ana.herrero@ictan.csic.es (A.M.H.); fjimenez@ictan.csic.es (F.J.-C.)

**Keywords:** meat species, free amino acid, biogenic amines, quality index

## Abstract

This paper studies the changes that occur in free amino acid and biogenic amine contents of raw meats (beef, pork, lamb, chicken and turkey) during storage (2 °C, 10 days). The meat cuts samples were harvested from a retail outlet (without getting information on the animals involved) as the following: Beef leg (four muscles), pork leg (five muscles), lamb leg (seven muscles), turkey leg (four muscles), and chicken breast (one muscle). Meat composition varied according to meat types. In general, pH, microbiology counts, biogenic amine (BA), and free amino acid (FAA) contents were also affected by meat types and storage time (*p* < 0.05). Chicken and turkey presented the highest levels (*p* < 0.05) of FAAs. Total free amino acids (TFAA) were higher (*p* < 0.05) in white meats than in red ones. The behavior pattern, of the total free amino acids precursors (TFAAP) of Bas, was saw-toothed, mainly in chicken and turkey meat during storage, which limits their use as quality indexes. Spermidine and spermine contents were initially different among the meats. Putrescine was the most prevalent BA (*p* < 0.05) irrespective of species. In general, chicken and turkey contained the highest (*p* < 0.05) levels of BAs, and TFAAP of BAs. In terms of the biogenic amine index (BAI), the quality of chicken was the worst while beef meat was the only sample whose quality remained acceptable through the study. This BAI seems to be more suitable as a quality index for white meat freshness than for red meat, especially for beef.

## 1. Introduction

**Figure 1:**
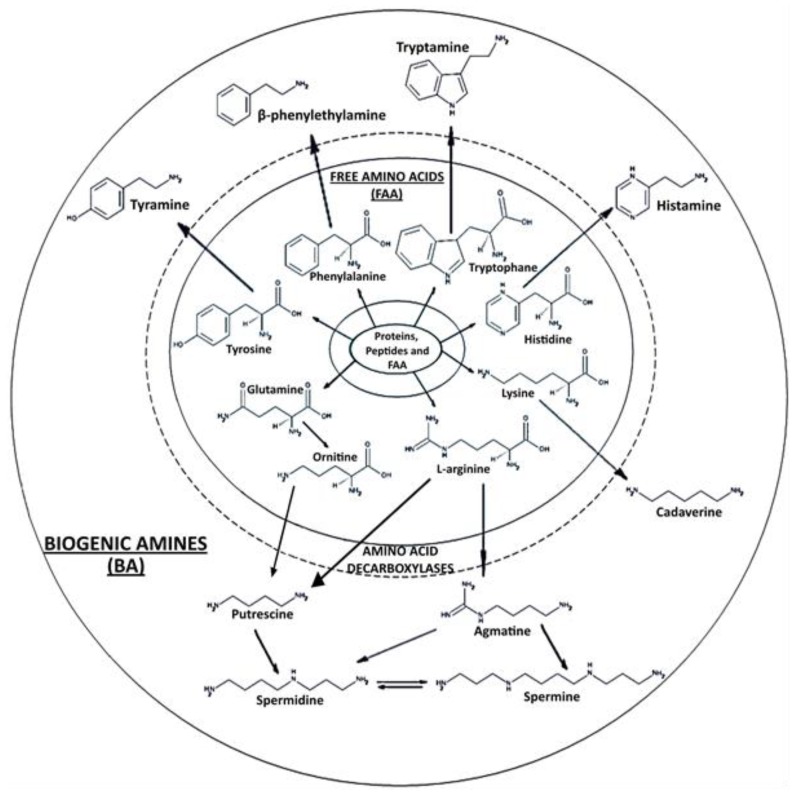
Biogenic amine formation from free amino acids.

Meat and meat products constitute an important protein group of foods, that can be consumed directly or as products after undergoing different processes. Consumers nowadays are asking for safe and high quality meat products. This quality is influenced by various factors, and complex interactions between the biological traits of the live animal, including mainly the biological processes that occur postmortem as muscles conversion to meat, processing and storage phases, etc. [1,2]. These meat and meat products, especially when they are fresh, undergo spoilage even during refrigerated storage [3]. This deterioration is associated with major proteolysis and microbial growth. Due to proteolysis, peptides, dipeptides, and free amino acids (FAA) are formed and used by microorganisms for their growth. Following these processes, different compounds, including biogenic amines (BA), are formed by amino acid decarboxylase action from microbial origin (Figure 1). The biogenic amine content depends on a number of interrelated factors such as the raw material (meat composition, pH, handling and hygienic conditions, etc.), additives (salt, sugar, nitrites), etc. These factors affect free amino acid availability, microbiological aspects (bacterial species and strain, bacterial growth, etc.), technical processing of the meat or meat products (e.g., steaks, roasts and hams, and ground, restructured, comminuted, fresh, cooked, smoked, and fermented meats, etc.), and storage conditions (time/temperature, packaging, temperature abuse, etc.). The combined action of all these factors will determine the final biogenic amine profile and concentrations by directly or indirectly determining substrate and enzyme presence and activity.

Therefore, biogenic amines are of particular concern in food hygiene. Indeed, they have been used as quality indices, mainly in fish and meat, under different processing and storage conditions, whether considered individually or in combined forms [4,5,6,7,8,9]. In this regard, Hernández-Jover et al. [10] suggested a biogenic amine index (BAI) as a sum of tyramine, histamine, putrescine and cadaverine, with four-scale classification intended for cooked meat products which were based on the sum of total BA concentration in the BAI. Tyramine is also widely used individually as a quality indicator for vacuum-packed beef and cooked ham; this is also the case for spermidine and spermine [11]. Other authors as Vinci & Antonelli [12] proposed the use of cadaverine and tyramine concentrations to assess beef and chicken deterioration during storage. Moreover, some BAs were studied for their potential toxicity for consumers, especially tyramine, histamine, cadaverine and putrescine [5,13,14]. Based on these considerations, regulations have been introduced to limit BA intake levels in various kinds of food [15,16].

Consequently, the determination of biogenic amines and free amino acid fractions can provide useful information for the industry regarding freshness or spoilage and sanitary quality of fresh muscle that could be consumed directly or used as raw material for meat products preparation. High concentrations of certain amines in food may be interpreted as a consequence of poor quality of the raw materials used, contamination, or inappropriate conditions during food processing and storage. In this regard, numerous studies were carried out in order to understand how FAA and biogenic amine formation is associated with microorganisms development in various meat products, mainly in fermented ones [5,7,8,17,18], and rarely in fresh meat [12]. On the other hand, “already-formed BAs” in the raw meat materials cannot be destroyed by thermal action during meat product processing or cooking, and this can lead to higher amine levels at the end of the pool [5,9]. However, some authors reported that BA formation not only depends on the conservation method used (refrigeration, protective atmosphere, etc.) or the type of processing (fermented, cooked, fresh, etc.), but it also depends on the type of raw material or animal species studied. Delgado-Pando et al. [19] and Triki et al. [20,21] observed differences in BA levels in reformulated (pork) frankfurters and fresh (beef) “merguez” sausages. Thus, FAA and BA production and microbial growth in fresh meat is of great interest for understanding and controlling the influence of raw meat as a factor in the final quality of fresh meat products (hamburger, fresh sausages, etc.). This approach might improve the safety as well as hygiene aspects of raw meat whether when they are used in the preparation for other meat products or employed for direct consumption or during the chilled storage of such foodstuffs. The aim of this study is, then, to assess the changes that take place in free amino acid and biogenic amine contents during chilled storage of fresh meats from some of the most frequently consumed species (beef, pork, lamb, chicken and turkey), which are used in the preparation of meat products as raw material.

## 2. Material and Methods

### 2.1. Fresh Meat Samples

Approximately 4 kg of commercial cuts of each type of fresh lean muscle meat from five species were purchased from a local supermarket. Leg cuts were taken from the four species: Beef (*Rectus femoris* M., *Semitendinosus* M., *Flexor digitorum longus* M., *Gastrocnemius* M.), pork (*Biceps femoris* M., *Semimembranosus* M., *Semitendinosus* M., *Gracilis* M., *Adductor* M.), turkey (*Flexor perforans* M., *Gastrocnemius pars external* M., *Gastrocnemius pars internal* M., *Fiburalis longus* M.), and lamb (*Quadriceps femoris* M., *Biceps femoris* M., *Semimembranosus* M., *Gluteus medius* M., *Gastrocnemius* M., *Adductor* M., *Semitendinosus* M.). Breast cuts were taken from chicken (*Pectoralis Major*). Two hundred to two hundred and fifty grams of each type of meat cut were representative of the pieces. Then meat cuts were placed on expanded polystyrene (EPS) trays (Type 89 white SPT-Linpac Packaging Pravia, S.A., Pravia, Spain) and covered with oxygen-permeable cling film (LINPAC Plastics, Pontivy, France) in aerobic conditions. From the 15 trays of each meat type that were kept in chilled storage (0 to 4 °C), three were taken periodically for further analysis. First was taken the sample for the microbiological analysis and then the sample was homogenized for the other analysis (protein content, pH, FAA and BA). Samples were assessed at 0, 3, 6, and 10 days of chilled storage.

### 2.2. Protein Content and pH Determination

Protein content was measured in quadruplicate with a LECO FP-2000 Nitrogen Analyzer (Leco Corporation, St. Joseph, MI, USA). For pH determination, 10 g homogenate samples in 100 mL of distilled water were prepared using a pH meter (827 pH Lab Methrom, Herisau, Switzerland). Both analyses followed the methodology used by Triki et al. [20]. Three measurements were performed per sample.

### 2.3. Microbiological Analysis

Ten grams of each representative sample were taken and placed in a sterile plastic bag with 90 mL of peptone water (0.1%) with 0.85% NaCl. After 2 min in a stomacher blender (Stomacher Colworth 400, Seward, UK), appropriate decimal dilutions were pour-plated (1 mL) on the following media: Plate Count Agar (PCA) (Merck, Darmstadt, Germany) for the total viable count (TVC) (30 °C for 72 h); De Man, Rogosa, Sharpe Agar (MRS) (Merck, Darmstadt, Germany) for lactic acid bacteria (LAB) (30 °C for 3–5 days); and Violet Red Bile Glucose Agar (VRBG) (Merck, Darmstadt, Germany) for *Enterobacteriaceae* (37 °C for 24 h). All microbial counts were converted to logarithms of colony-forming units per gram (Log cfu/g), following the methodology used by Triki et al. [20].

### 2.4. Determination of Free Amino Acids (FAA)

Free amino acids extracts were prepared with 5 g of fresh meat samples from each species. They were homogenized with 10 mL of perchloric acid 6% (*w*/*v*) (to extract the FAA and precipitate the proteins and peptides) in an Ultraturrax homogenizer (IKA-Werke, Janke, & Kunkel, Staufen, Germany) then centrifuged at 27,000× *g* (Sorvall RTB6000B, DuPont, Wilmington, DE, USA) for 10 min at 4 °C. 2 mL of KOH 1M were added to the centrifugation tube and the whole was centrifuged again. Afterwards, the supernatant was filtered through a Millipore filter (45 µm) (Millipore, Ireland) and put into vials until use [6].

Free amino acids (FAA) were determined by cation-exchange chromatography, using a Biochron 20 automatic amino acid analyser (Amersham Pharmacia LKB, Biotech Biocom, Uppsala, Sweden) with an Ultropac high-resolution cation-exchange resin column (9 ± 0.5 μm particle size, Pharmacia, Biotech) 200 × 4.6 mm. Amino acids were determined and measured using a ninhydrin derivative reagent at 570 nm, while proline was measured at 440 nm. It should be noted that the derivatization used did not allow the determination of tryptophan. Results are means of at least three determinations.

Total FAA precursors (TFAAP) of BA was calculated by summing the levels of the following FAA: Tyrosine + Phenylalanine + Histidine + Lysine + Arginine

Total FAA (TFAA) was calculated by summing the levels of the following FAAs: Aspartic acid + Threonine+ Serine + Glutamic acid + Glycine + β alanine + Cysteine + Valine + Methionine + Isoleucine + Leucine + TFAAP

### 2.5. Determination of Biogenic Amines (BA)

Tyramine, phenylethylamine, histamine, putrescine, cadaverine, tryptamine, agmatine, spermidine, and spermine were determined using an acid based extraction prepared with trichloroacetic acid (7.5%) in fresh meat samples from each specie. They were analyzed in a HPLC model 1022 with a Pickering PCX 3100 post-column system (Pickering Laboratories, Mountain View, Ca, USA) following the methodology of Triki et al. [22] by ion-exchange chromatography. Briefly, 15 g of/from each sample were mixed with 30 mL of 7.5% trichloroacetic acid in an omnimixer (Omni Internacional, Waterbury, CT, USA) (20,000 rpm, 3 min) and centrifuged at 5000 g for 15 min at 4 °C in a desktop centrifuge (Sorvall RTB6000B, DuPont, Wilmington, DE, USA) (for proteins and peptides precipitation and BA extraction in the supernatant). The supernatants were filtered through a Whatman No. 1 filter, passed back through a 0.22 µm Nylon filter (Millipore, Ireland), and then placed in opaque vials in the auto-sampler of the HPLC. The results are averages of at least 3 determinations.

Biogenic amine index (BAI) was calculated by summing tyramine, histamine, putrescine and cadaverine levels in the different meat types according to Hernández-Jover et al. [10]. When one of the BA involved was not detected (ND), its value was considered as being 0. BAI < 5 mg/kg means good meat quality; between 5–20 mg/kg means acceptable meat quality; between 20–50 mg/kg means poor meat quality; and BAI > 50 mg/kg means spoiled meat.

### 2.6. Statistical Analysis

A One-way ANOVA analysis of variance was performed in order to evaluate the statistical significance (*p* < 0.05) of the meat type effect. Analysis of the main effect of each independent variable and any interaction between them were carried out with two-way ANOVA, which was performed as a function of meat type and storage days, using SPSS Statistics general linear model (GLM) procedure (v.14, SPSS Inc.; Chicago, IL, USA). The types of meat and storage time were assigned as fixed effects and the replication (samples were taken from meat types of different animals) was considered as a random effect. Least squares differences were used for comparison between the mean values among meat types and Tukey’s HSD (honestly significant difference) test was used to identify significant differences (*p* < 0.05) between sample type and storage time. The error terms used throughout this study are standard deviations (SD). For the presented tables throughout the study: Different superscript letters in the same row indicate significant difference (*p* < 0.05) between storage days for the same meat type. Different superscript numbers in the same column indicate significant difference (*p* < 0.05) between meat types for the same storage day.

## 3. Results and Discussion

### 3.1. Protein Content and pH

Protein contents of meat (pork, 21.30 ± 0.16; lamb 19.32 ± 0.39; beef, 21.78 ± 0.25; turkey 20.02 ± 0.21; chicken 25.55 ± 0.36) were within the normal ranges for each species and type of meat cut [23]. Differences between protein amounts were due to the nature of species and cuts. As reported previously, protein is an important precursor of various compounds involved in BA formation.

The initial pH levels were significantly higher in pork (6.70) followed by turkey, chicken, lamb and beef (Table 1). This variance could be due to post mortem metabolism difference between species. As a matter of fact, both intrinsic (species, animal age, type of muscle and position of the muscle, concentration of glycogen etc.) and extrinsic (pre-slaughter stress, slaughter conditions, post-slaughter handling and temperature) factors can affect the extent of post-mortem glycolysis, and consequently the ultimate pH [1,24].

Initial pH levels for fresh beef and lamb meats were considered normal in comparison with reported levels in the literature [25,26]. However, they were high in pork, turkey and chicken compared with other studies [27,28,29]. These initial values were related to the kind of the cut for each species and its quality according to the rigor’s resolution. The pH increased in all meat types during storage, except for pork, and at the end of the storage they were between 6.01 and 7.34 (Table 1). These results agree with previous reports in different types of meats, also during refrigerated storage [30]. The increases were associated with the production of nitrogenized basic compounds, mainly aminic, which are the main results of microbial spoilage and are conditioned by the type of packaging. On the other hand, some studies reported lower pH values particularly for pork, turkey and chicken [31].

### 3.2. Microbiology

Microbial growth (TVC, LAB and *Enterobaceteriaceae*) was affected by meat type and storage time (*p* < 0.05) (Table 2). At the beginning of the experiment, the highest level of TVC was observed in lamb (5.64 Log cfu/g) and the lowest in chicken (4.13 Log cfu/g). Similar behavior was observed in LAB counts. Initial levels of *Enterobacteriaceae* were the lowest in pork (2.95 Log cfu/g). Generally, similar amounts were also reported by other authors for meat and meat products based on the different meat types [17,20,21,32,33].

Microbial growth (TVC, LAB, *Enterobacteriaceae*) increased by 1 and 2 logarithmic units during refrigerated storage (Table 2). After three days, all meat samples registered 6 Log cfu/g of TVC. These levels reached more than 8.9 Log cfu/g on day six and up to one more Log cfu/g unit until day 10. Other authors reported similar microbial behavior during chilled storage of fresh meat [17,20,21]. TVC values are commonly associated with meat spoilage when it reaches levels higher than 6 Log cfu/g [34]. The high levels reported during the experiment were in relation of the high pH levels of the samples (Table 1).

Microorganisms levels in raw meat are influenced by many factors which directly affect meat quality such as animal stress susceptibility, pre- and post-slaughter handling, processing, transport, packaging, storage, composition, etc. The experiment could have been finalized at microorganism levels that limit meat consumption, but samples were analyzed for a longer period in order to better understand BA and FAA formation and the relationship between microbial counts, FAA, and BA levels.

### 3.3. Free Amino Acids (FAA)

**Figure 2:**
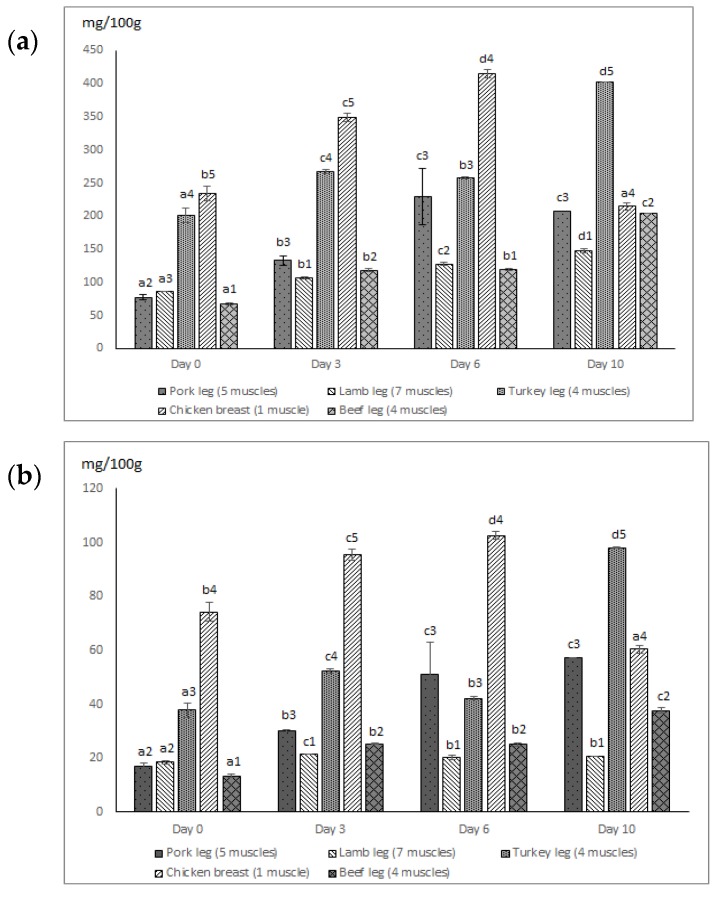
Total free amino acid (TFAA) (**a**) and Total free amino acid precursors (TFAAP) (**b**) of biogenic amines in mg/100 g of the different meat types during chilled storage at 2 °C. (Each value is the mean of three replicates per meat sample and storage day ± standard deviation (SD). Different letters indicate significant difference (*p* < 0.05) between storage days for the same meat type and different numbers indicate significant difference (*p* < 0.05) between meat types for the same storage day).

During storage, free amino acid levels and their behavior varied considerably depending on the type of meat (Table 3 and Figure 2).

Initially, the most abundant FAA in all meat samples was β-alanine, with levels of 18.70–37.64 mg/100 g, followed by the glutamic acid (4.42–28.95 mg/100 g) (Table 3). High levels of glycine, threonine and aspartic acid were also detected at the beginning of the storage. The lowest amounts were observed in cysteine, while histidine was not detected in any sample. Serine was highly present in turkey and chicken (37.63 and 27.67 mg/100 g, respectively) but it is not present in pork as much as in the aforementioned meat types (5.18 mg/100 g). However, it was detected neither in lamb nor beef until the sixth and tenth days of storage respectively (Table 3).

In general, chicken and turkey presented the highest levels of total free amino acids (TFAA) and TFAAP of Bas, while the lowest levels were observed in lamb which are followed by beef and pork (Figure 2a,b). These differences seem to be related to the type of meat (poultry or mammals). Indeed, white meats (chicken and turkey) registered approximately twice the amounts of TFAAs (234.30 and 201.33 mg/100 g respectively) as lamb (86.56 mg/100 g) and three times more than pork (77.50 mg/100 g) or beef (67.5 mg/100 g) at the beginning of storage (Figure 2a). These results are consistent with reports by the USDA [35] and other authors such as Leggio et al. [18], who reported levels between 47.5 and 93.07 mg/kg of TFAA of industrial “sopressata” pork sausage. Even though higher levels have been reported in chicken [36], low concentrations were found by Cowieson et al. [37] and Rabie et al. [7] in chicken, and in other meats (horse, beef, and turkey).

These differences could be due to many variables possibly influencing the formation and destruction of FAAs in the various meat types. These include factors such as the intrinsic properties of the product (biological factors relating to species, breed, sex, etc.; physiological aspects—genetic background, stress responses, etc.; and production practice—feeding, finishing weight, age at slaughter, etc.), handling, and processing conditions, etc. [5,38].

In general, the relative initial differences in TFAAs and TFAA precursors of BAs in chicken and turkey were maintained until the end of storage. Contents were at their highest (*p* < 0.05) in these species, although they were smaller in chicken than in turkey at the end of the experiment following a decrease after day six (Figure 2a,b). Glutamic acid was the most prevalent FAA at the end of the storage (reaching 87.58 mg/100 g in turkey and 65.41 mg/100 g in beef), followed by β-alanine (48.33 mg/100 g in turkey and 41.50 mg/100 g in lamb), and lysine (27.13 mg/100 g in chicken and 60.33 mg/100 g in turkey), which is a precursor of cadaverine. Serine registered a considerable decrease in all samples while valine, methionine and isoleucine increased from the initial levels (Table 3). The concentration of histidine, a precursor of histamine, was very low or beneath the threshold of detection throughout the study since it is not typically present in meat, but it is rather one of the characteristics of fish products [6]. On the other hand, Arginine was the most prevalent FAA precursor at the beginning of the storage and went undetected at its end. This decrease in arginine was due to agmatine and putrescine formation, which can also lead to spermidine and spermine production since the formation of these three amines is interrelated [13,39].

Some meat types (mainly chicken and turkey) presented a saw-toothed pattern for TFAA and TFAAP of BA over storage (Figure 2a,b). The saw-toothed pattern of the FAAs observed during the experiment is typical of the one reported in myosystems such as meat [35] and in various research studies on fish and seafood [40]. This pattern is related to both formation and destruction of FAAs [6,41], which are associated with meat proteolysis (breakdown of proteins into small peptides and free amino acids) during storage. This hydrolysis of the peptide bonds may be of endogenous (endogenous proteolytic enzymes as exopeptidases) or exogenous origin. The latter origins are associated with microbial activity and the transformation of FAAs into other compounds through chemical and metabolic reactions. Other authors [36] also reported significant increases of amino acid levels in chicken during refrigerated storage that were associated with proteolysis. The saw-toothed pattern could limit the use of FAAs as reliable quality indexes for fresh refrigerated meat, as reported elsewhere [6].

Nevertheless, the presence of certain amino acids such as glutamic acid, β-alanine, and phenylalanine were associated with typical flavors in meat and myosystems [42]. They are also very important as potential flavor and odor precursors through their interactions during heating, which contributes to the flavor and/or odor of cooked meat [43]. Indeed, glutamic acid and glycine are used as flavor enhancing additives in the food industry [44,45].

### 3.4. Biogenic Amines

Biogenic amine contents of the different meat types were affected (*p* < 0.05) by storage (Table 4). Except for the physiological amines spermidine and spermine (Spd and Spm), the initial levels of BAs were very low, and in some cases they were not detected. Spermidine and spermine presented the highest (*p* < 0.05) concentrations of BAs at the beginning of the experiment in all meat types, with spermine as the most abundant one (27.60–45.03 mg/kg). Levels of Spd and Spm were significantly lower in pork and beef as opposed to the rest of the meats, with the highest levels detected in chicken. The reported amounts of these amines in the literature are wide-ranging. Similar values of Spd and Spm were reported in meat products formulated with pork and beef [5,7,12,20,21,22,39], in raw turkey [46], lamb, and sheep liver [47]. On the other hand, Rokka et al. [48] reported higher levels of Spm and Spd in chicken in comparison with our results, while other authors observed smaller amounts for Spm [12,32]. In addition, significantly lower levels of the physiological amines were observed in lamb and mutton as in our study [47].

During refrigerated storage, the levels of physiological amines fluctuated in turkey for Spd and in turkey and chicken for Spm following a saw-toothed pattern (Table 4). Irrespective of the meat type. Both amines increased, peaking after the third day (18.27 mg/kg of Spd in turkey and 53.60 mg/kg of Spm in chicken) which was followed by a decrease until the end of the storage. This pattern, which is reported in other meat products in chilled storage [7], could reflect the relationship between these FAAs and the evolution pattern of their precursor, arginine (Figure 1). Initial arginine levels were considerable (Table 3). In fact, these levels increased up to day six and then decreased. Finally, they disappeared in all the samples except in turkey, which registered very low levels at the end of the experiment.

Putrescine (Put) was also detected throughout the study in all samples. Initial levels of these physiological amines were low in pork (0.57 mg/kg) while the other meat types contained the double of that amount (Table 4). Put levels increased significantly in all meats during the experiment but not at the same rate for all samples. For instance, in lamb and turkey, levels increased considerably by day three but the highest Put levels were recorded in chicken and turkey at the end of the study (51.99 mg/kg and 68.72 mg/kg, respectively). These were the highest levels among all BAs, even higher than tyramine, which is the most prevalent BA in meat products. At the end of the storage, only in the case of pork and lamb, similar levels of putrescine and tyramine were observed (Table 4). These putrescine levels were mainly related to *Enterobacteriaceae* growth (Table 2), which registered the highest levels. In addition, put production is associated with a reduction in arginine, which was also observed in all samples (Table 3) as noted earlier. Put is identified as one of the toxic biogenic amines, together with cadaverine (Cad), since they favor intestinal absorption of HIS and Tyr and contribute to catabolism reduction, thus enhancing their toxicity [5,14].

Cadaverine (Cad) is another important amine that was not detected in the meat samples until day six of chilled storage, except for beef, where it was not detected throughout the study. Other authors [20] reported that cadaverine was undetectable in beef whereas the meats in which it was detected, concentrations rose quickly, except for lamb. However, considerable levels were observed in pork, chicken, and turkey (16.16, 14.31 and 13.25 mg/kg, respectively). In most cases, no clear relationship was observed regarding the levels of its FAA lysine precursor, except for chicken and turkey, in which there was some correlation (coefficient > 0.92). It is worth noticing that at the end of storage, lysine was the precursor with the highest levels, peaking in turkey and chicken at day six of the experiment (Table 3). High levels of the FAA precursors that did not correlate with high levels of their corresponding BAs could be due to the lower BA decarboxylation capacity of the microorganisms that grow in this type of meat. These amines are also associated with *Enterobacteriaceae* growth; but, in this study, there was no clear relationship. However, there are other factors that can also affect BA presence in meat or meat products such as processing, meat matrix nature, etc. [9,20,21,22,49].

Agmatine (Agm) was present only in lamb meat with very low levels (0.15–2.30 mg/kg) (Table 4) starting from the third day of storage. However, its precursor arginine was widely represented in all meats, especially chicken, throughout the study (Table 3). This inverse behavior in lamb could be due to the weakness or lack of aminogenetic capacity of the microbiota [8] and the fact that arginine can be transformed into Putrescine (Figure 1) depending on the microbial flora type present in the matrix. As explained before, put levels were indeed consistent with the arginine levels observed in this study.

In general, the other BAs’ levels increased, from day three until the end of the storage. Levels of toxic amines such as tyramine (Tyr) and β-phenylethylamine (Phe) were very low (<0.8 mg/kg) at the beginning of the study and were not even detected in some meats such as in chicken and turkey (Tyr), and in pork and chicken (Phe). However, they were detected afterwards in all the studied kinds of meats during storage. These amounts increased during the experiment until day 10, but levels remained below 36 mg/kg in the case of Tyr, which reached its highest levels in chicken and lowest in beef (1.57 mg/kg), followed by turkey (6.88 mg/kg), lamb (10.71 mg/kg), and pork (16.58 mg/kg). Phe levels registered lower amounts in all species at the end of the storage. Chicken also contained the highest levels of Phe (12.81 mg/kg), followed by turkey, lamb, beef, and pork (Table 4) at the end of the experiment. These levels are very low in terms of toxicological limits, especially for Tyr (800 mg/kg) [50] while the limit for Phe is 30 mg/kg, which is twice the level found in this study [51]. These BAs are formed from the decarboxylation reactions of tyrosine and phenylalanine, respectively. The highest levels of tyrosine were observed in chicken, which can be associated with the high levels of tyramine in this species. However, no clear relationship was observed for the other species regarding tyramine and tyrosine even though the evolution of this FAA was clear throughout the storage and was species-dependent (Table 3). As a matter of fact, in some species, such as pork, lamb, beef or turkey, tyramine increased over storage, and these changes correlated with tyrosine production. On the other hand, the evolution in chicken followed a saw-toothed pattern, thus the possibility for establishing a correlation became more unlikely with Tyr production. Several authors reported similar evolutions of these FAAs and their relationship with BAs [6].

Phenylalanine levels (Table 3) were higher than tyrosine’s although its corresponding biogenic amine (Phe) levels were significantly lower than tyramine in some species. Phenylalanine levels were significantly higher at the end of the storage in all meat types, particularly turkey, chicken, and pork (Table 3). Other authors also found little correlation between Phe and its FAA precursor, which seems to be closely related to the nature of the flora and its aminogenic capacity [8,52]. Lamb, for example, contained higher levels of Phe (9.05 mg/kg) at the end of the experiment than of phenylalanine (5.82 mg/100 g) suggesting that its flora has high Phenylalanine decarboxylation capacity. The rest of the meats presented the opposite pattern suggesting that their flora presented less phenylalanine decarboxylation capacity.

On the other hand, histamine (HIS), another toxicological amine, was not detected in the majority of meat types (pork, lamb and turkey), except in chicken and beef where levels were very low (2.11 and 0.50 mg/kg, respectively at the end of storage) (Table 4). This was consistent with histidine levels, the FAA precursor of HIS (Table 3), which were not initially detected in any type of meat, except for pork and chicken, with very low levels (<6 mg/100 g). Final HIS levels in the meat types samples were significantly lower than the legal limit of 50 mg/kg [15], meaning that there is no potential health risk after consumption of these meats in their fresh status. Moreover, HIS levels are related to those of its FAA precursor, which is poorly represented in the studied meats (Table 3). Some authors also reported very low to undetectable HIS levels in fresh beef sausages and dry fermented pork sausages [20,21,39]. These low amounts are consistent with the type of samples analyzed since HIS is not typically found in fresh meat and meat products [5].

Tryptamine (Trp) was detected only in chicken and pork. While its presence in chicken was observed from the beginning of the storage, in the case of pork it was only detected after six days.

In general, higher BA levels (Table 4) were observed in chicken, turkey and pork, and these results were associated with the total free amino acid BA precursors (TFAAP) (Table 3). Given the importance of BAs as quality indexes, the application of the biogenic amines index (BAI) in this study showed a clear increase over storage, which was related to the meat type (Table 4). At the outset, all the BAIs registered less than 2 mg/kg, with the highest levels in chicken. According to the index’s classification, all meat types presented good quality, which was maintained until day six, afterwards there was a considerable increase in BAI levels, with the highest registered once more in chicken. The latter reached levels of 40.72 mg/kg, followed by pork (17.41 mg/kg), lamb (16.87 mg/kg), turkey (11.43 mg/kg), and beef (4.73 mg/kg). According to this index, chicken was considered of poor quality at day six while the other meats were still classified as acceptable. However, this classification is not related to microorganism levels on the same day of analysis (Table 2). As a matter of fact, a delay was observed in the formation of biogenic amines with respect to microbial growth. Such a delay was also reported by other authors [21,53] and constituted one of the factors that some authors used to set the limit of 6 Log/cfu for TVC as indicating unfitness for consumption.

At the end of storage, the highest BAI levels were observed in chicken (103.87 mg/kg) and turkey (88.85 mg/kg), which were spoiled, and the lowest BAI level was found in beef (9.47 mg/kg), which was still the only meat in the range of BAI acceptability (Table 4), while pork and lamb were considered as exhibiting poor quality.

Several authors demonstrated that beef is less spoilable than the other types of meats and that chicken is the first to undergo deterioration reactions [12,54]. BAI levels in turkey, beef, and pork presented a high correlation with TFAAP (0.97, 0.92, and 0.86, respectively) as well as with TFAA levels in lamb (0.97), beef (0.96, and turkey (0.95) throughout the storage (Table 4).

These results support the theory of rapid white meat spoilage compared with red meat [12,54], as shown in the FAA section. This behavior was also observed in tuna, where amine levels were generally higher in the white than in the red muscle [40]. In this study; the BAI levels of white meats (turkey and chicken) close to and above 50, at 10 days of chilling storage.

These BAI levels reflect the rate of deterioration of each type of meat and thus provide useful information when planning meat product processing and manufacture strategies. These BAI levels showed better results than some BA contents such as Tyramine or Spermidine and Spermine individually. However, BAI results did not show any clear relationship with microbial levels, which exceeded the permitted limits of microorganisms in all types of meats from day three of storage (Table 2). Therefore, in this case, the BAI index seems to be a more suitable indicator for white meat freshness than for red ones, especially beef, which was also reported by other authors [10].

## 4. Conclusions

The evolutions of free amino acids (FAA) and biogenic amines (BA) were clearly influenced by the meat type. The largest amounts were observed in chicken and turkey followed by the other meat types. Even though a clear relationship was observed for certain meat species between the biogenic amine index (BAI) and total free amino acids (TFAA), this index did not correlate with microbial growth. The relationship between overall TFAAs and BAIs was closer between an FAA precursor and its corresponding biogenic amine when considered individually. BAIs showed that only beef maintained acceptable quality throughout the study (<10 at day 10 of chilling storage), while chicken presented a poor quality (103.57) followed by turkey, lamb and pork. Overall, BA levels were higher in white meats than in red ones. During storage, some TFAAP of BA followed a saw-toothed pattern mainly in chicken and turkey meat. This limits its use as a quality index for fresh meat during chilled storage. Finally, the BAI index seems to be more suitable as a quality index for white meat freshness than for red meat, especially for beef.

## Figures and Tables

**Table 1:** pH values of the fresh different meat types during chilled storage at 2 °C.

Meat Type	Chilling Storage at 2 °C			
	Day 0	Day 3	Day 6	Day 10
Pork leg (5 muscles)	6.70 ± 0.02 ^c5^	5.89 ± 0.02 ^a2^	6.26 ± 0.03 ^b3^	6.32 ± 0.01 ^b3^
Lamb leg (7 muscles)	5.90 ± 0.01 ^b2^	6.05 ± 0.01 ^c3^	5.77 ± 0.01 ^a2^	6.01 ± 0.03 ^c1^
Turkey leg (4 muscles)	6.54 ± 0.01 ^a4^	6.89 ± 0.01 ^b5^	6.63 ± 0.02 ^a4^	7.34 ± 0.01 ^c5^
Chicken breast (1 muscle)	6.39 ± 0.26 ^b3^	6.30 ± 0.02 ^a, b4^	6.26 ± 0.01 ^a3^	6.67 ± 0.00 ^c4^
Beef leg (4 muscles)	5.71 ± 0.01 ^a1^	5.71 ± 0.10 ^a1^	5.52 ± 0.02 ^a1^	6.20 ± 0.01 ^b2^

Each value is the mean of three replicates per meat sample and storage day ± standard deviation (SD). Different superscript letters in the same row indicate significant difference (*p* < 0.05) between storage days for the same meat type. Different superscript numbers in the same column indicate significant difference (*p* < 0.05) between meat types for the same storage day.

**Table 2:** Microbial counts Log (cfu/g) of the fresh different meat types during chilled storage at 2 °C.

Microorganisms	Meat Type	Chilling Storage at 2 °C			
		Day 0	Day 3	Day 6	Day 10
Total Viable Count (TVC)	Pork leg (5 muscles)	5.53 ± 0.12 ^a4^	7.79 ± 0.05 ^b3^	9.74 ± 0.01 ^c4^	10.04 ± 0.01 ^d1, 2^
	Lamb leg (7 muscles)	5.64 ± 0.25 ^a4^	7.54 ± 0.40 ^b2^	9.99 ± 0.17 ^c5^	10.04 ± 0.02 ^c2^
	Turkey leg (4 muscles)	4.98 ± 0.01 ^a3^	6.45 ± 0.13 ^b1^	8.96 ± 0.05 ^c1^	9.91 ± 0.09 ^d1^
	Chicken breast (1 muscle)	4.13 ± 0.17 ^a1^	7.79 ± 0.03 ^b3^	9.55 ± 0.02 ^c3^	10.04 ± 0.05 ^d1, 2^
	Beef leg (4 muscles)	4.74 ± 0.10 ^a2^	6.55 ± 0.09 ^b1^	9.25 ± 0.04 ^c2^	10.22 ± 0.03 ^d2^
Lactic acid bacteria (LAB)	Pork leg (5 muscles)	3.76 ± 0.07 ^a3^	5.64 ± 0.01 ^b3^	7.57 ± 0.03 ^c5^	8.34 ± 0.08 ^d2^
	Lamb leg (7 muscles)	4.24 ± 0.01 ^a4^	5.70 ± 0.05 ^b3^	6.05 ± 0.11 ^c3^	8.26 ± 0.09 ^d2^
	Turkey leg (4 muscles)	4.65 ± 0.03 ^a5^	5.61 ± 0.10 ^b3^	7.40 ± 0.02 ^c4^	8.08 ± 0.13 ^d1^
	Chicken breast (1 muscle)	2.96 ± 0.02 ^a1^	5.38 ± 0.06 ^b2^	6.26 ± 0.02 ^b1^	8.99 ± 0.09 ^c1^
	Beef leg (4 muscles)	3.20 ± 0.14 ^a2^	4.34 ± 0.06 ^b1^	5.89 ± 0.01 ^c2^	8.04 ± 0.06 ^d1^
*Enterobacteriaceae*	Pork leg (5 muscles)	2.95 ± 0.00 ^a1^	4.95 ± 0.04 ^b1^	6.11 ± 0.05 ^c1^	7.53 ± 0.10 ^d1^
	Lamb leg (7 muscles)	4.71 ± 0.00 ^a4^	6.55 ± 0.05 ^b3, 4^	6.37 ± 0.02 ^b3^	7.99 ± 0.04 ^c2^
	Turkey leg (4 muscles)	4.24 ± 0.02 ^a3^	5.88 ± 0.06 ^b2^	6.79 ± 0.02 ^c4^	7.68 ± 0.03 ^d4^
	Chicken breast (1 muscle)	4.07 ± 0.76 ^a2, 3^	6.73 ± 0.03 ^b4^	6.72 ± 0.04 ^b2^	7.27 ± 0.09 ^c1^
	Beef leg (4 muscles)	3.92 ± 0.03 ^a2^	6.42 ± 0.12 ^b3^	6.62 ± 0.01 ^c2^	7.66 ± 0.05 ^d3^

Each value is the mean of three replicates per meat sample and storage day ± standard deviation (SD). For every type of microorganism: Different superscript letters in the same row indicate significant difference (*p* < 0.05) between storage days for the same meat type, and different superscript numbers in the same column indicate significant difference (*p* < 0.05) between meat types for the same storage day.

**Table 3:** Free amino acids (FAA) concentration (mg/100 g) of the fresh different meat types during chilled storage at 2 °C.

	FAA	Meat Type	Chilling Storage at 2 °C			
			Day 0	Day 3	Day 6	Day 10
FAA no precursors of BA	Aspartic acid	Pork leg (5 muscles)	2.96 ± 0.04 ^a2^	6.82 ± 0.86 ^c3^	9.46 ± 2.59 ^d2^	4.87 ± 0.02 ^b1^
		Lamb leg (7 muscles)	1.72 ± 0.00 ^a1^	1.80 ± 0.31 ^a1^	4.00 ± 0.12 ^b1^	5.98 ± 0.04 ^c2^
		Turkey leg (4 muscles)	7.95 ± 0.41 ^a3^	13.61 ± 0.32 ^b4^	20.68 ± 0.02 ^d3^	15.92 ± 0.39 ^c3^
		Chicken breast (1 muscle)	15.38 ± 0.83 ^b3^	23.87 ± 0.16 ^c5^	29.18 ± 0.29 ^d4^	4.57 ± 0.11 ^a1^
		Beef leg (4 muscles)	2.37 ± 1.00 ^a1, 2^	5.15 ± 0.18 ^b2^	3.99 ± 0.00 ^b1^	4.96 ± 0.11 ^b1^
	Threonine	Pork leg (5 muscles)	2.21 ± 0.04 ^a2^	4.23 ± 0.23 ^b3^	11.23 ± 2.71 ^c3^	12.30 ± 0.04 ^c2^
		Lamb leg (7 muscles)	2.67 ± 0.04 ^a b2^	2.10 ± 0.02 ^a1^	4.08 ± 0.05 ^b c2^	4.97 ± 0.19 ^c1^
		Turkey leg (4 muscles)	8.30 ± 0.80 ^a3^	11.06 ± 0.48 ^b4^	12.40 ± 0.18 ^b3^	24.41 ± 0.14 ^c3^
		Chicken breast (1 muscle)	13.71 ± 0.59 ^a4^	21.17 ± 0.34 ^b5^	30.82 ± 0.23 ^c4^	12.51 ± 0.26 ^a2^
		Beef leg (4 muscles)	1.40 ± 0.04 ^a1^	2.58 ± 0.07 ^b2^	2.75 ± 0.07 ^b1^	5.10 ± 0.10 ^c1^
	Serine	Pork leg (5 muscles)	5.18 ± 0.45 ^a1^	9.12 ± 0.74 ^b1^	10.51 ± 2.41 ^c2^	8.45 ± 0.06 ^b3^
		Lamb leg (7 muscles)	ND	ND	5.84 ± 0.12 ^b1^	4.36 ± 0.00 ^a1^
		Turkey leg (4 muscles)	37.63 ± 1.04 ^c3^	41.59 ± 0.32 ^d2^	31.69 ± 0.17 ^b3^	15.26 ± 0.19 ^a4^
		Chicken breast (1 muscle)	27.67 ± 1.42 ^b2^	41.41 ± 0.52 ^d2^	36.99 ± 0.73 ^c4^	8.85 ± 0.28 ^a3^
		Beef leg (4 muscles)	ND	ND	ND	7.40 ± 0.04 ^a2^
	Glutamic acid	Pork leg (5 muscles)	8.09 ± 0.96 ^a2^	16.78 ± 0.94 ^b3^	48.67 ± 1.93 ^d3^	28.97 ± 0.19 ^c1^
		Lamb leg (7 muscles)	5.94 ± 0.14 ^a1^	11.05 ± 0.26 ^b2^	19.09 ± 0.39 ^c2^	27.66 ± 1.73 ^d1^
		Turkey leg (4 muscles)	28.95 ± 1.66 ^a4^	50.35 ± 0.09 ^b5^	55.64 ± 0.04 ^b4^	87.58 ± 0.19 ^c3^
		Chicken breast (1 muscle)	21.07 ± 0.88 ^a3^	36.66 ± 0.54 ^c4^	51.58 ± 0.73 ^d3^	30.52 ± 0.66 ^b1^
		Beef leg (4 muscles)	4.42 ± 0.38 ^a1^	8.81 ± 0.08 ^b1^	15.06 ± 0.30 ^c1^	65.41 ± 0.14 ^d2^
	Glycine	Pork leg (5 muscles)	6.17 ± 0.37 ^a2^	12.65 ± 0.77 ^b c2^	13.71 ± 3.14 ^c2^	11.23 ± 0.06 ^b2^
		Lamb leg (7 muscles)	18.10 ± 0.02 ^a4^	19.89 ± 0.06 ^b3^	17.88 ± 0.33 ^a3^	17.69 ± 0.42 ^a3^
		Turkey leg (4 muscles)	18.24 ± 0.99 ^a4^	20.88 ± 0.22 ^b3,4^	18.62 ± 0.18 ^a3^	28.28 ± 0.01 ^c4^
		Chicken breast (1 muscle)	16.76 ± 0.75 ^b3^	21.93 ± 0.44 ^c4^	27.41 ± 0.45 ^d4^	11.74 ± 0.21 ^a2^
		Beef leg (4 muscles)	3.14 ± 0.07 ^a1^	10.31 ± 0.08 ^c1^	6.73 ± 0.18 ^b1^	7.04 ± 0.03 ^b1^
	β-alanine	Pork leg (5 muscles)	18.70 ± 1.06 ^a1^	29.68 ± 1.51 ^b1^	38.51 ± 8.70 ^c2^	27.69 ± 0.03 ^b1^
		Lamb leg (7 muscles)	30.83 ± 0.00 ^a3^	36.65 ± 0.09 ^c2^	32.65 ± 0.68 ^b1^	41.50 ± 0.87 ^d2^
		Turkey leg (4 muscles)	37.64 ± 2.14 ^a4^	42.34 ± 0.10 ^b3^	41.90 ± 0.45 ^b2^	48.33 ± 0.11 ^c3^
		Chicken breast (1 muscle)	22.68 ± 0.86 ^a2^	38.59 ± 0.67 ^c2^	52.54 ± 0.87 ^d3^	28.75 ± 0.72 ^b1^
		Beef leg (4 muscles)	29.09 ± 0.20 ^a3^	47.69 ± 0.34 ^c4^	41.59 ± 0.77 ^b2^	42.99 ± 0.30 ^b2^
	Cysteine	Pork leg (5 muscles)	0.53 ± 0.12 ^a2^	0.92 ± 0.04 ^b2^	1.61 ± 0.27 ^c3^	1.71 ± 0.03 ^c3^
		Lamb leg (7 muscles)	0.20 ± 0.04 ^a1^	1.05 ± 0.09 ^b2^	1.17 ± 0.00 ^b2^	1.20 ± 0.04 ^b1^
		Turkey leg (4 muscles)	0.33 ± 0.00 ^a1^	0.39 ± 0.00 ^a1^	0.42 ± 0.04 ^a1^	2.01 ± 0.09 ^b4^
		Chicken breast (1 muscle)	0.22 ± 0.00^a1^	0.33 ± 0.00 ^a1^	3.95 ± 0.04 ^c4^	1.74 ± 0.09 ^b3^
		Beef leg (4 muscles)	0.98 ± 0.04 ^a3^	0.98 ± 0.12 ^a2^	1.06 ± 0.00 ^a2^	1.42 ± 0.03 ^b2^
	Valine	Pork leg (5 muscles)	5.77 ± 0.04 ^a2^	8.17 ± 0.45 ^b3^	13.16 ± 2.74 ^c3^	16.25 ± 0.06 ^d3^
		Lamb leg (7 muscles)	2.59 ± 0.08 ^a1^	4.64 ± 0.08 ^b1^	7.58 ± 0.14 ^c1^	7.09 ± 0.11 ^c1^
		Turkey leg (4 muscles)	8.29 ± 0.37 ^a3^	11.28 ± 0.43 ^b4^	11.49 ± 0.18 ^b2^	23.19 ± 0.14 ^c4^
		Chicken breast (1 muscle)	12.97 ± 0.47 ^a4^	20.28 ± 0.34 ^c5^	23.45 ± 0.18 ^d4^	16.67 ± 0.61 ^b3^
		Beef leg (4 muscles)	5.30 ± 0.08 ^a2^	6.23 ± 0.56 ^b2^	8.26 ± 0.16 ^c1^	9.37 ± 0.04 ^d2^
	Methionine	Pork leg (5 muscles)	2.67 ± 0.29 ^a2^	3.17 ± 0.10 ^a2^	9.31 ± 1.94 ^b3^	12.30 ± 0.04 ^c3^
		Lamb leg (7 muscles)	1.10 ± 0.00 ^a1^	1.44 ± 0.30 ^a1^	3.97 ± 0.01 ^b1^	4.12 ± 0.10 ^b1^
		Turkey leg (4 muscles)	3.19 ± 0.07 ^a2^	4.84 ± 0.34 ^b3^	5.00 ± 0.04 ^b2^	16.79 ± 0.01 ^c4^
		Chicken breast (1 muscle)	7.10 ± 0.30 ^a3^	11.58 ± 0.27 ^b4^	14.63 ± 0.21 ^c4^	12.34 ± 0.29 ^b3^
		Beef leg (4 muscles)	1.31 ± 0.00 ^a1^	2.20 ± 0.68 ^a1, 2^	4.09 ± 0.05 ^b1^	6.62 ± 0.08 ^c2^
	Isoleucine	Pork leg (5 muscles)	2.98 ± 0.12 ^a3^	4.36 ± 0.25 ^b2^	8.28 ± 1.74 ^c2^	9.88 ± 0.08 ^d3^
		Lamb leg (7 muscles)	1.73 ± 0.13 ^a1^	2.40 ± 0.10 ^b1^	3.92 ± 0.08 ^c1^	4.05 ± 0.00 ^c1^
		Turkey leg (4 muscles)	5.25 ± 0.21 ^a4^	7.24 ± 0.18 ^b3^	6.97 ± 0.08 ^b2^	16.33 ± 0.00 ^c4^
		Chicken breast (1 muscle)	8.16 ± 0.35^a5^	13.98 ± 0.23 ^c4^	15.75 ± 0.40 ^d3^	10.08 ± 0.20 ^b3^
		Beef leg (4 muscles)	2.21 ± 0.04 ^a2^	3.02 ± 0.51 ^a b1^	3.90 ± 0.05 ^b1^	5.88 ± 0.07 ^c2^
	Leucine	Pork leg (5 muscles)	5.35 ± 0.08 ^a2^	6.88 ± 0.38 ^b3^	14.18 ± 2.92 ^c3^	16.65 ± 0.06 ^d3^
		Lamb leg (7 muscles)	3.09 ± 0.34 ^a1^	4.20 ± 0.14 ^b1^	7.05 ± 0.19 ^c1^	7.22 ± 0.04 ^c1^
		Turkey leg (4 muscles)	7.66 ± 0.40 ^a3^	11.28 ± 0.10 ^b4^	10.84 ± 0.07 ^b2^	26.19 ± 0.06 ^c4^
		Chicken breast (1 muscle)	14.29 ± 0.44 ^a4^	24.10 ± 0.46 ^c5^	26.76 ± 0.46 ^d4^	16.86 ± 0.60 ^b3^
		Beef leg (4 muscles)	3.79 ± 0.04 ^a1^	5.65 ± 0.54 ^b2^	6.99 ± 0.05 ^c1^	9.99 ± 0.09 ^d2^
FAA precursors of BA	Tyrosine	Pork leg (5 muscles)	3.82 ± 0.24 ^a3^	5.02 ± 0.35 ^b3^	7.90 ± 1.79 ^c3^	8.94 ± 0.27 ^c3^
		Lamb leg (7 muscles)	2.13 ± 0.06 ^a2^	2.36 ± 0.04 ^b1^	2.62 ± 0.17 ^c1^	3.09 ± 0.47 ^c1^
		Turkey leg (4 muscles)	6.21 ± 0.34 ^a4^	8.75 ± 0.13 ^b4^	8.20 ± 0.41 ^b3^	9.24 ± 0.07 ^a3^
		Chicken breast (1 muscle)	11.23 ± 0.54 ^b5^	18.14 ± 0.43 ^d5^	15.80 ± 0.20 ^c4^	17.17 ± 0.27 ^c4^
		Beef leg (4 muscles)	1.63 ± 0.18 ^a1^	2.96 ± 0.17 ^b2^	5.22 ± 0.18 ^c2^	6.04 ± 0.56 ^d2^
	Phenylalanine	Pork leg (5 muscles)	3.94 ± 0.33 ^a3^	4.39 ± 0.37 ^a3^	14.82 ± 3.31 ^b3^	17.41 ± 0.22 ^b3^
		Lamb leg (7 muscles)	2.36 ± 0.22 ^a2^	2.50 ± 0.12 ^a1^	4.86 ± 0.63 ^b1^	5.82 ± 0.59 ^b1^
		Turkey leg (4 muscles)	4.10 ± 0.46 ^a3^	6.87 ± 0.28 ^b4^	6.45 ± 0.15 ^b2^	19.92 ± 0.05 ^c4^
		Chicken breast (1 muscle)	8.24 ± 0.12 ^a4^	13.70 ± 0.35 ^b5^	15.74 ± 0.34 ^c3^	18.41 ± 0.78 ^d3^
		Beef leg (4 muscles)	1.72 ± 0.27 ^a1^	3.54 ± 0.06 ^b2^	4.72 ± 0.22 ^c1^	13.18 ± 0.00 ^d2^
	Histidine	Pork leg (5 muscles)	ND	3.26 ± 0.16 ^a1^	3.85 ± 0.91 ^a1^	5.00 ± 0.03 ^b1^
		Lamb leg (7 muscles)	ND	ND	ND	ND
		Turkey leg (4 muscles)	ND	ND	ND	ND
		Chicken breast (1 muscle)	ND	ND	ND	5.62 ± 0.78 ^a1^
		Beef leg (4 muscles)	ND	ND	ND	ND
	Lysine	Pork leg (5 muscles)	4.96 ± 0.29 ^a1^	9.92 ± 056 ^b1^	24.46 ± 5.63 ^c3^	25.84 ± 0.04 ^c3^
		Lamb leg (7 muscles)	7.69 ± 0.00 ^a3^	9.64 ± 0.06 ^b1^	12.69 ± 0.16 ^c1^	12.76 ± 0.24 ^c1^
		Turkey leg (4 muscles)	15.09 ± 0.92 ^a4^	20.66 ± 0.17 ^b3^	26.19 ± 0.26 ^c3^	60.33 ± 0.04 ^d5^
		Chicken breast (1 muscle)	32.38 ± 1.74 ^b5^	37.50 ± 0.73 ^c4^	69.68 ± 0.89 ^d4^	27.13 ± 0.52 ^a4^
		Beef leg (4 muscles)	5.91 ± 0.05 ^a2^	12.09 ± 0.17 ^b2^	13.58 ± 0.16 ^c2^	18.56 ± 1.63 ^d2^
	Arginine	Pork leg (5 muscles)	4.19 ± 0.40 ^b1^	7.56 ± 0.58 ^c1^	0.12 ± 0.01 ^a2^	ND
		Lamb leg (7 muscles)	6.39 ± 0.28 ^b2^	7.06 ± 0.12 ^c1^	0.08 ± 0.01 ^a1^	ND
		Turkey leg (4 muscles)	12.49 ± 0.89 ^b3^	16.17 ± 0.13 ^c2^	1.45 ± 0.23 ^a3, 4^	0.72 ± 0.04 ^d5^
		Chicken breast (1 muscle)	22.44 ± 1.21 ^b4^	26.32 ± 0.62 ^c3^	1.41 ± 0.06 ^a3^	ND
		Beef leg (4 muscles)	4.27 ± 0.11 ^b1^	6.74 ± 0.33 ^c1^	1.57±0.06 ^a4^	ND
TFAAP of BA		Pork leg (5 muscles)	16.91 ± 1.25 ^a2^	30.15 ± 0.55 ^b3^	51.15 ± 11.76 ^c3^	57.18 ± 0.04 ^c3^
		Lamb leg (7 muscles)	18.58 ± 0.56 ^a2^	21.56 ± 0.02 ^c1^	20.25 ± 0.85 ^b1^	20.68 ± 0.12 ^b1^
		Turkey leg (4 muscles)	37.89 ± 2.61 ^a3^	52.46 ± 0.70 ^c4^	42.29 ± 0.52 ^b3^	90.21 ± 0.27 ^d5^
		Chicken breast (1 muscle)	74.29 ± 3.60 ^b4^	95.66 ± 2.12 ^c5^	102.63 ± 1.48 ^d4^	62.71 ± 1.37 ^a4^
		Beef leg (4 muscles)	13.53 ± 0.61 ^a1^	25.33 ± 0.27 ^b2^	25.09 ± 0.62 ^b2^	37.79 ± 1.07 ^c2^
TFAA		Pork leg (5 muscles)	77.50 ± 4.35 ^a2^	132.94 ± 6.63 ^b3^	229.78 ± 42.86 ^c3^	207.48 ± 0.23 ^c3^
		Lamb leg (7 muscles)	86.56 ± 0.10 ^a3^	106.78 ± 0.98 ^b1^	127.48 ± 2.46 ^c2^	147.51 ± 3.50 ^d1^
		Turkey leg (4 muscles)	201.33 ± 10.70 ^a4^	267.32 ± 3.10 ^c4^	257.94 ± 1.62 ^b3^	394.50 ± 0.36 ^d5^
		Chicken breast (1 muscle)	234.30 ± 10.49 ^b5^	349.56 ± 6.08 ^c5^	415.68 ± 6.08 ^d4^	217.34 ± 5.40 ^a4^
		Beef leg (4 muscles)	67.54 ± 0.94 ^a1^	117.96 ± 2.37 ^b2^	119.51 ± 2.12 ^b1^	203.98 ± 0.26 ^c2^

ND: Not Detected. Each value is the mean of three replicates per meat sample and storage day ± standard deviation (SD). For every type of free amino acid: Different superscript letters in the same row indicate significant difference (*p* < 0.05) between storage days for the same meat type, and different superscript numbers in the same column indicate significant difference (*p* < 0.05) between meat types for the same storage day.

**Table 4:** Biogenic amines (BA) concentration (mg/kg) of fresh different meat types during chilled storage at 2 °C.

BA	Meat Type	Chilling Storage at 2 °C			
		Day 0	Day 3	Day 6	Day 10
Tyramine	Pork leg (5 muscles)	0.67 ± 0.03 ^a3^	1.10 ± 0.02 ^b3^	11.20 ± 0.04 ^c4^	16.58 ± 0.04 ^d4^
	Lamb leg (7 muscles)	0.10 ± 0.00 ^a1^	0.19 ± 0.01 ^a1^	7.05 ± 0.25 ^b3^	10.71 ± 0.01 ^c3^
	Turkey leg (4 muscles)	ND	0.40 ± 0.04 ^a2^	1.72 ± 0.02 ^b2^	6.88 ± 0.14 ^c2^
	Chicken breast (1 muscle)	ND	0.47 ± 0.07 ^a2^	27.54 ± 0.86 ^b5^	35.16 ± 0.36 ^c5^
	Beef leg (4 muscles)	0.34 ± 0.04 ^a2^	0.42 ± 0.02 ^a2^	0.53 ± 0.03 ^b1^	1.57 ± 0.07 ^c1^
Histamine	Pork leg (5 muscles)	ND	ND	ND	ND
	Lamb leg (7 muscles)	ND	ND	ND	ND
	Turkey leg (4 muscles)	ND	ND	ND	ND
	Chicken breast (1 muscle)	0.53 ± 0.03 ^a1^	1.23 ± 0.03 ^b2^	1.73 ± 0.03 ^c2^	2.11 ± 0.01 ^d2^
	Beef leg (4 muscles)	ND	0.10 ± 0.00 ^a1^	0.21 ± 0.01 ^b1^	0.50 ± 0.02 ^c1^
Phenylethylamine	Pork leg (5 muscles)	ND	0.77 ± 0.03 ^a1^	1.28 ± 0.02 ^b1^	1.66 ± 0.06 ^c1^
	Lamb leg (7 muscles)	0.76 ± 0.00 ^a3^	4.80 ± 0.08 ^b3^	7.57 ± 0.27 ^c3^	9.05 ± 0.15 ^d3^
	Turkey leg (4 muscles)	0.21 ± 0.05 ^a1^	15.07 ± 0.01 ^d4^	12.85 ± 0.47 ^c4^	11.33 ± 0.29 ^b4^
	Chicken breast (1 muscle)	ND	16.87 ± 0.13 ^c5^	17.99 ± 0.15 ^b5^	12.81 ± 0.05 ^a5^
	Beef leg (4 muscles)	0.47 ± 0.01 ^a2^	2.33 ± 0.05 ^b2^	2.47 ± 0.05 ^b2^	2.62 ± 0.02 ^c2^
Putrescine	Pork leg (5 muscles)	0.57 ± 0.09 ^a1^	0.72 ± 0.02 ^a1^	5.10 ± 0.48 ^b2^	14.55 ± 0.09 ^c3^
	Lamb leg (7 muscles)	1.19 ± 0.07 ^a2^	3.22 ± 0.00 ^b4^	6.40 ± 0.24 ^c3^	10.11 ± 0.01 ^d2^
	Turkey leg (4 muscles)	1.23 ± 0.03 ^a2^	4.70 ± 0.08 ^b5^	8.44 ± 0.44 ^c5^	68.72 ± 0.02 ^d5^
	Chicken breast (1 muscle)	1.23 ± 0.03 ^a2^	1.83 ± 0.01 ^b2^	7.47 ± 0.07 ^c4^	51.99 ± 0.29 ^d4^
	Beef leg (4 muscles)	1.34 ± 0.02 ^a3^	2.07 ± 0.03 ^b3^	3.99 ± 0.03 ^c1^	7.40 ± 0.04 ^d1^
Cadaverine	Pork leg (5 muscles)	ND	ND	1.11 ± 0.07 ^a1^	16.16 ± 0.28 ^b4^
	Lamb leg (7 muscles)	ND	ND	3.42 ± 0.02 ^a2^	5.08 ± 0.12 ^b1^
	Turkey leg (4 muscles)	ND	ND	1.27 ± 0.03 ^a1^	13.25 ± 0.27 ^b2^
	Chicken breast (1 muscle)	ND	ND	3.98 ± 0.10 ^a3^	14.31 ± 0.11 ^b3^
	Beef leg (4 muscles)	ND	ND	ND	ND
Tryptamine	Pork leg (5 muscles)	ND	ND	6.37 ± 0.07 ^a1^	6.56 ± 0.10 ^a2^
	Lamb leg (7 muscles)	ND	ND	ND	ND
	Turkey leg (4 muscles)	ND	ND	ND	ND
	Chicken breast (1 muscle)	3.82 ± 0.12 ^b1^	15.78 ± 0.16 ^d1^	7.47 ± 0.07 ^c2^	0.37 ± 0.01 ^a1^
	Beef leg (4 muscles)	ND	ND	ND	ND
Agmatine	Pork leg (5 muscles)	ND	ND	ND	ND
	Lamb leg (7 muscles)	ND	0.15 ± 0.01 ^a1^	1.14 ± 0.02 ^b1^	2.30 ± 0.08 ^c1^
	Turkey leg (4 muscles)	ND	ND	ND	ND
	Chicken breast (1 muscle)	ND	ND	ND	ND
	Beef leg (4 muscles)	ND	ND	ND	ND
Spermidine	Pork leg (5 muscles)	2.63 ± 0.13 ^a2^	2.70 ± 0.00 ^a1^	3.18 ± 0.14 ^b1^	3.88 ± 0.18 ^c1^
	Lamb leg (7 muscles)	8.09 ± 0.13 ^a4^	11.99 ± 0.13 ^c4^	8.69 ± 0.21 ^a4^	10.16 ± 0.12 ^b5^
	Turkey leg (4 muscles)	7.33 ± 0.05 ^a3^	18.27 ± 0.07 ^d5^	12.14 ± 0.10 ^c5^	9.67 ± 0.09 ^b4^
	Chicken breast (1 muscle)	9.78 ± 0.06 ^d5^	6.69 ± 0.13 ^b3^	6.24 ± 0.20 ^a3^	7.02 ± 1.10 ^c3^
	Beef leg (4 muscles)	2.29 ± 0.05 ^a1^	3.39 ± 0.05 ^b2^	4.35 ± 0.01 ^c2^	5.39 ± 0.05 ^d2^
Spermine	Pork leg (5 muscles)	27.60 ± 0.96 ^b1^	27.10 ± 0.12 ^b1^	25.23 ± 1.17 ^a1^	26.87 ± 0.31 ^b2^
	Lamb leg (7 muscles)	31.36 ± 0.58 ^a2^	40.85 ± 0.37 ^c3^	31.60 ± 0.98 ^a3^	36.42 ± 0.00 ^b4^
	Turkey leg (4 muscles)	35.44 ± 1.28 ^b3^	49.20 ± 0.40 ^d4^	36.95 ± 0.53 ^c4^	32.55 ± 0.09 ^a3^
	Chicken breast (1 muscle)	45.03 ± 0.81 ^b4^	53.60 ± 0.24 ^d5^	47.26 ± 0.54 ^c5^	41.92 ± 0.16^a5^
	Beef leg (4 muscles)	30.86 ± 0.36 ^b2^	33.02 ± 0.24 ^c2^	29.54 ± 0.40 ^b2^	25.08 ± 0.04 ^a1^
BAI	Pork leg (5 muscles)	1.24	1.82	17.41	47.29
	Lamb leg (7 muscles)	1.29	3.41	16.87	25.90
	Turkey leg (4 muscles)	1.23	5.10	11.43	88.85
	Chicken breast (1 muscle)	1.76	3.53	40.72	103.57
	Beef leg (4 muscles)	1.68	2.59	4.73	9.47

Each value is the mean of three replicates per meat sample and storage day ± standard deviation (SD). ND: Not detected (average limit of detection being 0.065 mg/L). For every biogenic amine: Different superscript letters in the same row indicate significant difference (*p* < 0.05) between storage days for the same meat type, and different superscript numbers in the same column indicate significant difference (*p* < 0.05) between meat types for the same storage day.
